# Parental feeding practices in Mexican American families: initial test of an expanded measure

**DOI:** 10.1186/1479-5868-10-6

**Published:** 2013-01-17

**Authors:** Jeanne M Tschann, Steven E Gregorich, Carlos Penilla, Lauri A Pasch, Cynthia L de Groat, Elena Flores, Julianna Deardorff, Louise C Greenspan, Nancy F Butte

**Affiliations:** 1 Department of Psychiatry, University of California at San Francisco, Box 0848, San Francisco, CA, 94143-0848, USA; 2 Department of Medicine, University of California at San Francisco, Box 0856, San Francisco, CA, 94143-0856, USA; 3 Counseling Psychology Department, School of Education, University of San Francisco, 2130 Fulton Street, San Francisco, CA, 94118, USA; 4 Division of Community Health and Human Development, School of Public Health, 50 University Hall, University of California at Berkeley, Berkeley, CA, 94720-7360, USA; 5 Kaiser Permanente, 2200 O'Farrell Street, San Francisco, CA, 94115, USA; 6 Baylor College of Medicine, USDA/ARS Children's Nutrition Research Center, Department of Pediatrics, 1100 Bates Street, Houston, TX, 77030-2600, USA

**Keywords:** Feeding practices, Mexican Americans, Latinos, Child weight, Child obesity, Mothers, Fathers, Parent–child relationships, Scale development

## Abstract

**Background:**

Although obesity rates are high among Latino children, relatively few studies of parental feeding practices have examined Latino families as a separate group. Culturally-based approaches to measurement development can begin to identify parental feeding practices in specific cultural groups. This study used qualitative and quantitative methods to develop and test the Parental Feeding Practices (PFP) Questionnaire for use with Mexican American parents. Items reflected both parent’s use of control over child eating and child-centered feeding practices.

**Methods:**

In the qualitative phase of the research, 35 Latino parents participated in focus groups. Items for the PFP were developed from focus group discussions, as well as adapted from existing parent feeding practice measures. Cognitive interviews were conducted with 37 adults to evaluate items. In the quantitative phase, mothers and fathers of 174 Mexican American children ages 8–10 completed the PFP and provided demographic information. Anthropometric measures were obtained on family members.

**Results:**

Confirmatory factor analyses identified four parental feeding practice dimensions: positive involvement in child eating, pressure to eat, use of food to control behavior, and restriction of amount of food. Factorial invariance modeling suggested equivalent factor meaning and item response scaling across mothers and fathers. Mothers and fathers differed somewhat in their use of feeding practices. All four feeding practices were related to child body mass index (BMI) percentiles, for one or both parents. Mothers reporting more positive involvement had children with lower BMI percentiles. Parents using more pressure to eat had children with lower BMI percentiles, while parents using more restriction had children with higher BMI percentiles. Fathers using food to control behavior had children with lower BMI percentiles.

**Conclusions:**

Results indicate good initial validity and reliability for the PFP. It can be used to increase understanding of parental feeding practices, children’s eating, and obesity among Mexican Americans, a population at high risk of obesity.

## Background

Obesity rates among children in the US are high and increasing [1]. Obesity among Mexican American children is of particular concern. Among children 6–11 years old, 22% of Mexican American girls and 27% of Mexican American boys were obese in 2007–2008, compared with 17% of non-Hispanic white girls and 21% of non-Hispanic white boys [1]. As a result, there is a critical need to identify modifiable risk factors for obesity in this population. One important influence on children’s weight, for which interventions could be developed, is parental feeding practices. Parental feeding practices are thought to influence children’s weight status indirectly, through children’s eating behavior and nutritional intake [2]. The literature on self-reported parental feeding practices is growing, but relatively few studies have focused specifically on Latino families [3-8]. The purpose of the current study was to develop and validate a culturally appropriate measure of parental feeding practices for use with Mexican American families.

The majority of studies examining parental feeding practices have focused on parental use of control in child feeding, such as restriction of food and pressure to eat. Such practices may impede children’s ability to self-regulate their food intake, by causing children to focus on external cues instead of their own hunger and satiety [9]. Restricting foods may increase their desirability [10], while pressure to eat appears to reduce the desirability of the food being offered [11,12]. Other feeding practices that reflect parental control are feeding in response to emotional distress (emotional regulation) and using food as a reward (instrumental feeding). These two feeding practices are also thought to override children’s internal cues regarding hunger and satiety and lead to negative eating patterns [13]. Finally, parental monitoring of children’s intake of unhealthy foods has been characterized both as parental control in child feeding [14] and as evidence of positive parenting [15].

In contrast to parental use of control in child feeding, child-centered feeding practices are hypothesized to promote healthy eating and protect against obesity. Child-centered feeding practices are thought to reflect authoritative parenting, a general parenting style in which parents are both responsive to children’s needs and demanding of maturity [16,17]. Child-centered feeding practices may allow children to develop self-regulation in response to their own internal cues of hunger and satiety [5]. Such feeding practices include offering children healthy food, allowing them to eat the amount they desire, reasoning with children, complimenting, and encouraging balance and variety [5,18,19].

One goal of the current study was to develop a measure of self-reported parental feeding practices that assesses a broad range of feeding practices encompassing both parental use of control and child-centered feeding practices. Within the past decade, several measures of parental feeding practices have been developed. However, most of these measures do not incorporate the full range of potentially important feeding practices. For example, the widely-used Child Feeding Questionnaire (CFQ) focuses primarily on parental use of control in feeding, including pressure to eat and restriction of food [20]. The Caregiver’s Feeding Styles Questionnaire (CFSQ: [5,21]) and the Parental Feeding Style Questionnaire (PFSQ: [13]) focus on parental control over feeding as well as child-centered feeding practices, but do not assess a wide range of either type of feeding practice. The Comprehensive Feeding Practices Questionnaire (CFPQ: [19]) provides the most complete assessment of both parental control and child-centered feeding practices.

A second goal of our research was to use qualitative methods to produce a culturally appropriate measure of parental feeding practices in Mexican American families. It is not yet clear the degree to which some aspects of parental feeding practices are culturally specific. Two studies suggest that Hispanic parents’ self-reported restriction include somewhat different behaviors than those of non-Hispanic white parents [3,20] . Although initial factor analyses of the CFQ indicated that using food as a reward for good behavior is a type of restriction [20], reward and restriction appear to be two separate constructs for Hispanic parents [3]. In observational research, Latina mothers engaged in higher rates of nondirective feeding strategies such as suggestions and questions than they did directive feeding strategies such as commands or forced compliance [22]. However, compared with other ethnic groups, Hispanic parents report higher levels of restriction and pressure to eat [4].

Given the possibility that Latino parents engage in culturally-specific feeding practices, an important step in developing measures specific to this group is to use culturally-based qualitative methods to identify relevant constructs. Although researchers have called for culturally-based approaches to measure development, such techniques are infrequently used [23,24]. Accordingly, it is not surprising that most existing measures of parental feeding practices were not developed specifically for use with Latino populations. For example, the CFQ has been validated for use with Hispanics [3,20], but items were not selected based on information from Latino populations. Thus, although the scale can be used with Latino populations, it is not clear if the full range of Latino parents’ control in feeding is captured by the CFQ. In contrast, the first version of the CFSQ was based partially on videotaped observations of African American and Hispanic parental feeding strategies [21]. Nonetheless, as noted above, this measure assesses a narrow range of behaviors. Finally, the CFPQ [19], which assesses a broad range of feeding practices, was not developed for use with Latinos, nor has it been validated in Latino populations to date. Thus, none of the existing parental feeding practices measures both incorporate a broad range of behaviors and also were developed for use in Latino populations.

A third goal of our research was to address the fact that most measures of self-reported parental feeding practices assess a mixture of attitudes, beliefs, motivations, and behaviors [19,20]. In this study, we attempted to provide more clarity regarding parents’ behaviors, by focusing solely on self-reported feeding practices, rather than also assessing attitudes and cognitions about children’s eating.

A final goal of this study was to include both mothers and fathers in the research. Parental feeding practices have generally been examined in mothers, and relatively few studies have reported on fathers’ feeding practices [25-28]. Mothers are considered to have a central role in child feeding, but the results of these studies suggest that fathers’ feeding practices also influence children’s weight status.

To address the goals of this research, we used a combination of qualitative and quantitative methods to develop and validate a comprehensive measure of parental feeding practices for use with Mexican American mothers and fathers. In the developmental/qualitative phase of the research, we adapted items from existing measures of parental feeding practices; conducted focus groups and created new items; and evaluated the cultural appropriateness of all items through cognitive interviewing. In the quantitative phase of the research, we assessed the structure of the scale, using confirmatory factor analyses, followed by internal consistency reliabilities. We then examined similarities and differences between mothers’ and fathers’ feeding practices. Finally, we tested correlations between parental feeding practices and children’s weight status to establish initial predictive validity of the measure.

## Method

### Developmental phase

We developed the Parental Feeding Practices (PFP) Questionnaire as part of a study examining parental influences on obesity among Mexican American children. We first adapted subscales from several existing parental feeding measures: restriction, pressure, and monitoring [20]; emotion regulation, food as reward, child control, and encourage balance/variety [19]; child-centered feeding [5]; and three items regarding monitoring and limiting [29]. We reworded items as needed to reflect parents’ behavior, rather than cognitions, perceptions, or motivations.

Next, 35 Latino parents participated in four focus groups, which we conducted according to suggested guidelines [30]. Two focus groups were conducted with each gender; two groups were conducted in Spanish and two in English. Focus groups facilitators were bilingual, bicultural, and matched by gender to the participants. Participants were asked to describe what parents say or do to encourage their children to eat healthily; they also responded to questions about what parents say or do when they are concerned that their children are eating too much, eating too little, not eating enough vegetables or fruit, want too much dessert, or snack too much.

From the focus group discussions, we developed 24 parental feeding practice items, using wording similar to that of focus group participants as much as possible. We combined these new items with the items adapted from existing measures. Items were translated into Spanish or English and reviewed side-by-side by a bilingual committee. Translations were compared for equivalent meaning, and items were revised or decentered as needed [31]. Decentering is a process in which both languages are considered equally important, and either language may be altered to obtain linguistically equivalent items. Items were revised as necessary until group consensus regarding equivalence was attained.

As a final step, we conducted cognitive interviews in Spanish or English with 37 Mexican American adults. During these interviews, interviewers read each item aloud and respondents were asked to comment on meaning, relevance, and comfort level [32,33]. Items were revised iteratively as needed to improve clarity, acceptability, and relevance, until all problematic wording was resolved. Previous research suggests that mothers often misunderstand questionnaire items regarding feeding practices [34], a finding that underscores the importance of cognitive interviewing to assure validity. The final PFP contained 68 items, representing both parental control and child-centered feeding practices.

### Quantitative phase

#### Procedure

After developing the PFP, we conducted the quantitative phase of the research. The study was approved by the university and Kaiser Permanente Northern California institutional review boards. We recruited families from the membership lists of Kaiser Permanente Northern California, an integrated health care delivery organization. Parents were sent letters introducing the research, were telephoned, screened for eligibility, and invited to participate in the study. Bilingual research assistants obtained written parental informed consent and verbal child assent to participate in the research. All study materials had been developed in both Spanish and English, and interviews were conducted in the language of participants’ choice. Most parents chose to be interviewed in Spanish (71% of mothers, 69% of fathers). Research assistants interviewed family members individually in their homes, and recorded responses to the questionnaires in laptop computers. Interviews were not conducted privately, because many participants’ homes were small. Interviews lasted about 1.5 hours. Research assistants also measured family members’ height and weight. Each family member was reimbursed $70 for study participation.

#### Participants

Families were eligible if the mother was Mexican origin (born in the US or Mexico), the child was 8–10 years of age, and the child had no major illnesses. This narrow age range was selected, because there may be age-related differences in feeding practices across a wider age range. Families were eligible even if fathers did not participate in the research, but every effort was made to recruit all fathers. If the father did not reside in the same household as the mother and child, the primary father figure was identified (i.e., biological father living apart or residential parental figure) and recruited to participate. Of the 322 mothers and children participating in the study, 54% (n = 174) of fathers participated. The current report is based on 174 mother-father pairs who provided PFP responses. Most fathers were biological fathers living with the mothers (90%); the remainder were stepfathers (7%), or biological fathers living apart from the mothers (3%). Most parents were born in Mexico (74% mothers; 74% fathers). The majority of fathers were of Mexican heritage (85%), and the remaining fathers were other Latino heritage (12%), or other/mixed ethnicities (3%). Parents’ average education was slightly less than high school graduation (mothers: M = 10.94 years, SD = 3.72; fathers: M = 11.47 years, SD = 3.61). Most parents were employed (73% of mothers, 89% of fathers). Parents’ occupational status [35] ranged from unskilled worker (=1) to major professional (=9), with the average occupation being skilled worker (mothers: M = 3.52, SD = 2.21; fathers: M = 3.72, SD = 1.85). Most parents were overweight (body mass index [BMI] > = 25 and <30; 37% of mothers, 46% of fathers) or obese (BMI >30; 42% of mothers, 46% of fathers). Participating children were 50% female, ages 8–10 (M = 9.24 years, SD = .89), and 95% had been born in the U.S. Based on age- and gender-specific BMI percentiles (CDC NCHS website [36]), 20% of the children were overweight and 28% were obese.

#### Measures

Parents completed the 68-item Parental Feeding Practices (PFP) Questionnaire (Table 1). They were instructed to answer the questions with the study child in mind. All questions were worded in terms of frequency of behavior, and response options ranged from 1 to 5 (“never, sometimes, often, very often, always”).

**Table 1:** Factor loadings from first- and second-order strict invariance models, and Cronbach alphas

**Second-order factor**	**(α mothers, α fathers)**	**1st-order**	**2nd-order**
***First-order factor***	**(α mothers, α fathers)**	**model**	**model**
**#. Item**			
**Positive involvement in child eating**	**(.88, .91)**		
*Monitor/limit high-calorie foods*	(.84, .90)		.75
03. How often do you limit the amount of high-fat foods (fried foods, French fries) your child eats?		.58	.57
04. How often do you keep track of the sweets (candy, ice cream, cake, pies, pastries) your child eats?		.63	.63
10. How often do you encourage your child to eat healthy foods before less healthy ones?		.60	.60
14. How often do you keep track of the snack food (potato chips, Doritos, cheese puffs) that your child eats?		.70	.70
18. How often do you limit the amount of sweets (candy, ice cream, cake or pastries) that your child eats?		.67	.67
25. How often do you keep track of the high-fat foods (fried foods, French fries) that your child eats?		.72	.72
34. How often do you limit the amount of junk foods your child can eat?		.74	.75
35. How often do you keep track of the sugary drinks (soda/pop, Kool-Aid) your child drinks?		.72	.73
48. How often do you limit the amount of soda your child drinks?		.55	.55
62. How often do you restrict the amount of fattening food your child can eat?		.58	.57
*Encourage/compliment healthy eating*	(.76, .82)		.91
08. How often do you say something positive about the food that your child is eating?		.51	.52
15. How often do you tell your child how tasty a new food is?		.58	.58
19. How often do you reason with your child to get him/her to eat (for example, Milk is good for your health because it will make you strong)?		.54	.53
32. How often do you tell your child that healthy food tastes good?		.61	.62
39. How often do you compliment your child for eating food (for example, What a good boy! You're eating your vegetables)?		.60	.59
49. How often do you encourage your child to eat by arranging the food to make it more interesting (for example, making smiley faces on the pancakes)?		.43	.43
52. How often do you encourage your child to try to eat healthy foods such as vegetables?		.66	.67
63. How often do you keep track of the servings of fresh fruits and vegetables your child is eating?		.58	.56
*Encourage a variety of new foods*	(.70, .64)		.72
21. How often do you encourage your child to try new foods?		.75	.72
42. How often do you encourage your child to eat a variety of foods?		.67	.70
*Ask child what he/she ate* †	(.55, .74)		.56
45. How often do you ask your child what he/she ate during the day?		.64	.63
55. How often do you find out how much your child ate during the day?		.79	.80
*Provide small servings*	(.39, .50)		.56
44. How often do you give your child small servings of food at meals?		.50	.46
60. How often do you add small servings of new foods to your child's plate?		.61	.66
**Pressure to eat**	**(.86, .84)**		
*Tell child to eat all food on plate*	(.84, .81)		.87
05. How often do you try to make your child eat all of the food on his/her plate?		.58	.57
43. How often do you tell your child he/she has to finish eating before he/she can go play or do something else?		.53	.53
46. How often do you remind your child to finish eating?		.55	.55
56. How often do you tell your child to eat everything on the plate?		.81	.82
61. How often do you tell your child if he/she doesn't eat, he/she can't watch TV?		.55	.55
64. How often do you tell your child he/she can't leave the table until he/she finishes?		.75	.74
66. How often do you insist that your child eat his/her meal?		.71	.71
*Require child to eat even if not hungry*	(.72, .69)		.81
16. If your child says, I'm not hungry, how often do you try to get him/her to eat anyway?		.55	.59
26. If your child eats only a small amount, how often do you try to get him/her to eat more?		.79	.76
36. When he/she says he/she is finished eating, how often do you try to get your child to eat one more (two more, etc.) bites of food?		.62	.62
**Use of food to control behavior**	**(.78, .75)**		
*Use food to control emotions*	(.78, .72)		.71
09. How often do you give your child something to eat or drink if he/she is cranky or grumpy, even if you think he/she isn't hungry?		.50	.51
20. How often do you give your child something to eat or drink if he/she is bored, even if you think he/she isn't hungry?		.63	.64
31. How often do you give your child something to eat or drink if he/she is upset, even if you think he/she isn't hungry?		.64	.64
41. How often do you give your child something to eat or drink if he/she is sad, even if you think he/she isn't hungry?		.64	.62
51. How often do you give your child something to eat or drink to make him/her happy, even if you think he/she isn't hungry?		.66	.65
*Use food as reward*	(.65, .63)		.83
02. How often do you offer your child his/her favorite foods in exchange for good behavior?		.52	.52
12. How often do you offer sweets (candy, ice cream, cake, pastries) to your child as a reward for good behavior?		.56	.56
33. How often do you tell your child that he/she has to finish dinner if he/she wants a sweet?		.60	.60
53. How often do you tell your child if he/she finishes the meal, he/she can have a sweet or a soda?		.62	.62
**Restriction of amount of food**	**(.77, .70)**		
*Encourage child to eat less*	(.78, .72)		.76
24. How often do you encourage your child to eat less?		.75	.75
28. How often have you put your child on a diet to control his/her weight?		.49	.50
38. How often do you tell your child he's/she's eaten enough?		.72	.73
54. If your child eats more than usual at one meal, how often do you try to restrict his/her eating at the next meal?		.65	.65
*Allow child to control snacking and second servings*	(.64, .58)		−.57
01. How often do you let your child eat whatever he/she wants?		.44	.46
30. How often do you allow your child to eat snacks whenever he/she wants?		.58	.53
37. How often do you let your child have seconds?		.50	.47
50. If your child asks for a snack, how often do you give it to him/her?		.65	.70
59. How often does your child get his/her own snack without asking first?		.32	.33
*Limit eating between meals*‡	(.48, .38)		.71
07. How often do you keep your child from eating between meals?		.42	.38
13. How often do you limit how much your child can eat his/her favorite foods?		.36	.38
57. How often do you limit the number of snacks your child eats?		.60	.64
**N/A**	(.62, .61)		
*Allow child to choose meal menu*			
11. At dinner, how often do you let your child choose the foods he/she wants from what is served?		.54	n/a
17. How often do you ask your child what he/she wants for dinner?		.49	n/a
22. If your child does not like what is being served, how often do you make something else?		.46	n/a
65. How often do you try to serve the meals your child likes?		.66	n/a
**N/A**			
*Allow child to leave table without finishing meal*	(.64, .59)		
06. How often do you tell your child to leave whatever he/she doesn't want to eat?		.56	n/a
27. How often do you tell your child he/she doesn't have to eat something he/she doesn't like?		.54	n/a
40. How often do you allow your child to leave the table when he/she is full, even if your family is not done eating?		.38	n/a
47. How often do you let your child leave food on his/her plate?		.68	n/a

Note: Both first- and second-order factor models had cross-parental equality constraints imposed on all corresponding parameter estimates; Second-order factor loadings are underlined; † first-order factor 'Ask child what he/she ate' had a cross-loading = .35 on second-order factor 'Restriction of amount of food'; ‡ first-order factor 'Limit eating between meals*'* had a cross-loading = .50 on second-order factor 'Positive involvement in child eating'.

Trained research assistants measured height and weight using standard procedures [37,38]. Height and weight were measured in duplicate while the participant was wearing light indoor clothing and no shoes. Child’s body mass index (BMI) was calculated (BMI = weight(kg)/height(m)^2^) and converted to age- and gender-specific percentile scores using NCHS growth charts (CDC NCHS website [36]). Parents’ BMI was also calculated.

Demographic variables included child age in months, child gender, parents’ years of education, occupational status [35], and acculturation. Occupational status ranged from lowest (=1) to highest (=9). Acculturation was assessed using the Spanish and English Language Use subscales of the Bidimensional Acculturation Scale for Hispanics [39]. Items are scored from never (=1) to always (=5), and have good reliabilities (alphas = .88 - .94). Parents had higher acculturation scores in Spanish (mothers: M = 4.09, SD = 1.30; fathers: M = 4.01, SD = 1.10) than in English (mothers: M = 2.82, SD = 1.30; fathers: M = 2.95, SD = 1.12).

We compared mothers who were included in the current report (n = 174) to mothers who were excluded due to non-participation by the child’s father (n = 148). Mothers who were included were more likely to be in a two-parent home (Chi-square = 58.88, p < .0001). They were also more acculturated (English subscale t [319] = 2.68, p < .008), had more years of education (t [318] = 3.49, p < .001), had higher occupational status (t [290] = 2.61, p < .009), and had lower BMI scores (t [313] = 2.56, p < .02). The two groups of mothers did not differ on parental feeding practices scores.

### Statistical analyses

The Additional file 1: Appendix describes the psychometric analysis plan in detail. We fit oblique principal components cluster analysis models (SAS PROC VARCLUS) of the 68 PFP items to initially identify a first-order factor structure. The fit of the first-order factor model and its equivalence across mothers and fathers was subsequently assessed with two confirmatory factor analysis (CFA) models: a configural invariance and a strict invariance model. The first-order configural invariance model imposed the same configuration of items and factors across mothers and fathers, but did not impose any equality constraints on corresponding parameter estimates for mothers and fathers. The first-order strict invariance model imposed equality constraints on corresponding parameter estimates for mothers and fathers, including factor loadings, item intercepts, and item residual variances, as well as factor variances and covariances. Following, to identify a second-order factor structure, we used summated scale scores representing each first-order factor (item cluster) as input data for an exploratory factor analysis with oblique rotation. Finally, we assessed the fit of the second-order factor model by fitting second-order configural invariance and strict invariance models via CFA. The second-order strict invariance model imposed equality constraints on corresponding parameters for mothers and fathers, including first- and second-order factor loadings, item and first-order factor intercepts, item and first-order factor residual variances, as well as second-order factor variances and covariances.

Although <1% of all data values were missing, we accommodated missing values via multiple imputation (MI) and the expectation-maximization (EM) algorithm [40-42]. We fit VARCLUS and EFA models to the EM item (or scale) covariance matrix and fit each CFA model to 5 multiply imputed data sets (PROC MI [43]) using LISREL 9.0 beta version [44]. For each CFA model, we report the Satorra-Bentler scaled chi-square statistic [45], root mean square error of approximation (RMSEA [46]), and comparative fit index (CFI [47]) values that were averaged across imputed data sets (see Additional file 1: Appendix). Generally, RMSEA values below .05 or .06 and CFI values above .95 suggest good approximate model fit [48,49].

Once a factor model was selected, we calculated first- and second-order scale scores by averaging non-missing item responses; resulting scores ranged from 1–5, and higher scores indicated higher frequency of the feeding practice. We next estimated internal-consistency reliabilities, inter-scale correlations, correlations between mothers’ and fathers’ scores, and correlations between feeding practices scales and children’s BMI percentiles.

## Results

### Factor analysis

We examined competing VARCLUS solutions and chose the 14-cluster model (listed as first-order factors in Table 1). Inspection of the VARCLUS solution led us to drop two items that shared little substantive content with the remaining items, and subsequent preliminary CFA modeling suggested dropping an additional three items because of relatively high cross-factor loadings or low primary factor loadings (see Additional file 1: Appendix); thus, 63 of the original 68 items were retained. The first-order configural and full invariance models provided good approximate fit: RMSEAs < .025 and CFIs > .96 (Table 2). The fit of the first-order configural invariance model suggested that the 14 clusters well-represented the inter-item associations among both mothers and fathers. The fit of the first-order strict invariance model further suggested that the meanings of the 14 clusters were equivalent across mothers and fathers and observed item (scale) means and variances can be meaningfully compared across mothers and fathers, i.e., observed scores of mothers and fathers are not subject to differential response biases [50-52]. The first-order strict invariance model factor loadings are listed in Table 1. Next, we explored second-order factor structures. EFA models that included all 14 first-order factor scale score suggested highly complex second-order factor structures that encountered estimation problems in subsequent CFA modeling. The second-order factor structure was greatly simplified after dropping two first-order factors: ‘Allow child to choose meal menu’ and ‘Allow child to leave table without finishing meal’. The resulting model had four second-order factors each for mothers and fathers. Both the second-order configural invariance and strict invariance models had good approximate fit: RMSEAs < .03 and CFIs > .95 (Table 2). These results suggest that the meanings of the second-order factors are equivalent for mothers and fathers and that the second-order scale score means and variances can be directly compared across mothers and fathers [50-52]. The first- and second-order factor loadings from the second-order strict invariance model are presented in Table 1.

**Table 2:** Model fit summary

**Model**	${\bar{\chi }}_{\mathrm{S-B}}^{2}$	**df**	**RMSEA**	**CFI**
1st-order configural invariance	7978.55	7308	.023	.968
1st-order strict invariance	8302.59	7574	.024	.965
2nd-order configural invariance	6561.95	5763	.028	.958
2nd-order strict invariance	6775.88	5942	.029	.956

### Internal consistency

The internal consistency of each first-order factor was evaluated separately for mothers and fathers, using Cronbach’s alpha (Table 1). Coefficients of most first-order factors were good for scales of these lengths, except for two factors with alphas < .50. The remaining coefficients ranged from .55 - .90, with an average value of .73. At the second-order factor level, alphas ranged from .70 - .91 and averaged .81.

### Correlations between second-order factor feeding practices scales

The inter-scale correlations at the second-order level are shown in Table 3. The largest correlations were between ‘Pressure to eat’ and ‘Use of food to control behavior’ (*r*s > .47, *p*s < .01). ‘Positive involvement in child eating’ was significantly correlated with ‘Pressure to eat’ and ‘Restriction of amount of food’ (*r*s = .23 - .37, *p*s < .01). (Correlations between the 14 first-order factors are shown in the Additional file 1: Appendix.)

**Table 3:** Correlations, means, and standard deviations of second-order feeding practices scales (mothers below the diagonal; fathers above the diagonal; correlations between mothers and fathers on the diagonal)

	**(1)**	**(2)**	**(3)**	**(4)**
Correlations				
(1) Positive involvement in child eating	.23*	.32*	.07	.37*
(2) Pressure to eat	.28*	.46*	.49*	−.04
(3) Use of food to control behavior	.05	.48*	.19*	−.03
(4) Restriction of amount of food	.23*	−.02	−.10	.45*
Means (SD)				
Mothers	3.58^a^ (.62)	2.38^b^ (.85)	1.50^c^ (.47)	2.77 (.59)
Fathers	3.37^a^ (.73)	2.55^b^ (.86)	1.60^c^ (.50)	2.76 (.57)

^*^p < .01.

^a, b, c^ Means sharing a common superscript are significantly different, *p* < .05.

### Similarities and differences between mothers’ and fathers’ feeding practices scores

Mothers’ and fathers’ feeding practices scores were modestly to moderately correlated (coefficients on the diagonal, Table 3). Mothers had higher scores than fathers on ‘Positive involvement in child eating’, while fathers had higher scores on ‘Pressure to eat’ and ‘Use of food to control behavior’ (Table 3).

### Correlations between parental feeding practices scales and children’s BMI

We first examined the correlations between demographic factors and children’s BMI percentiles, to identify demographics that should be used as control variables. Children with higher BMI scores had parents with higher Spanish language acculturation (mothers: *r* = .23, *p* < .002; fathers: *r* = .21, *p* < .007), lower occupational status (mothers: r = −.29, *p* < .0001; fathers: *r* = −.16, *p* < .04), and higher parental BMI (mothers: *r* = .31, *p* < .0001; fathers: *r* = .27, *p* < .0001). Children’s BMI scores were not significantly correlated with child age, child gender, parents’ years of education, or English language acculturation. When controlling for significant demographic factors, partial correlations showed that all parental feeding practices scales were related to children’s BMI percentiles, for one or both parents (Table 4). Mothers who reported more positive involvement in child eating had children with lower BMI scores. Parents who used more pressure to eat had children with lower BMI scores, while parents who used more restriction had children with higher BMI scores. Fathers who used food to control behavior had children with lower BMI scores. (Partial correlations between the first-order feeding practice scales and children’s BMI are shown in the Additional file 1: Appendix).

**Table 4:** Partial correlations between parental feeding practices scales and children’s BMI percentiles, controlling for parents’ acculturation, occupational status, and parents’ BMI

	**Children’s BMI**
Mothers’ feeding practices	
Positive involvement in child eating	-.18*
Pressure to eat	-.32***
Use of food to control behavior	-.09
Restriction of amount of food	.34***
Fathers’ feeding practices	
Positive involvement in child eating	-.04
Pressure to eat	-.33***
Use of food to control behavior	-.19*
Restriction of amount of food	.35***

* *p* < .05, ** *p* < .01, *** *p* < .001.

## Discussion

A growing literature examines parental feeding practices as a potential influence on children’s weight status. However, few studies of parental feeding practices have focused specifically on Mexican American children, in spite of the high rates of obesity in this population. Moreover, existing measures of parental feeding practices have either assessed a narrow range of feeding practices, or have not used culturally-based methodological approaches in measurement development. In the current study, we used a combination of qualitative and quantitative methods to develop and validate the Parental Feeding Practices (PFP) Questionnaire, an expanded measure of parental feeding practices in Mexican American families. Fathers as well as mothers participated in the research.

Confirmatory factor analysis revealed four second-order feeding practice dimensions: positive involvement in child eating, pressure to eat, use of food to control behavior, and restriction of amount of food. All four dimensions were equivalent in meaning for mothers and fathers. Within couples, mothers’ and fathers’ feeding practices were somewhat similar, but mothers and fathers differed in the degree to which they reported using some feeding practices. Moreover, each feeding practice dimension was related to child weight status, for one or both parents. For both parents, those who used more pressure to eat had children with lower BMI scores, while those who used more restriction had children with higher BMI scores. Mothers, but not fathers, who reported more positive involvement in their children’s eating had children with lower weight. In contrast, fathers who used food to control children’s behavior had children with lower weight status, but this association was not found for mothers.

Our study is among the first to provide evidence that mothers’ positive involvement in children’s eating is associated with children’s lower weight status. This constellation of behaviors, encompassing encouragement of healthy eating, monitoring and limiting intake of high-calorie foods, and serving small portions, is hypothesized to promote healthy eating and protect against obesity [9,18]. Positive involvement in children’s eating is thought to reflect authoritative parenting, a general parenting style that consists of parental demands for maturity and responsiveness to children’s needs [16,17]. Authoritative parenting has been linked to children’s and adolescents’ competence in a variety of domains, such as social interactions and academic success [53]. Recently, investigators have begun to examine whether general parenting styles, such as authoritative parenting, are reflected in specific parental feeding practices [15,21].

One aspect of parents’ positive involvement in their children’s eating that emerged in this research was parental food monitoring and limiting of high-calorie foods. Parental food monitoring/limiting has been conceptualized as parental control of child feeding by some investigators [14] and a constructive feeding practice by others [15]. In this study, parental food monitoring/limiting was linked with other feeding practices generally considered to be positive, and was distinct from restriction of amount of food. These findings suggest that parental monitoring/ limiting of high-calorie foods is a constructive feeding practice, at least among Mexican Americans. In future research with this and other populations, it would be useful to confirm that monitoring/limiting high-calorie foods and restricting amount of food are separate constructs.

Both pressure to eat and restriction of amount of food were associated with children’s weight. Consistent with previous research [6,20,54], mothers’ and fathers’ pressure to eat was related to children’s lower weight status. In contrast, mothers’ and fathers’ restricting amount of food was related to children’s higher weight status, consistent with some but not all previous research [55,56]. Both of these feeding practices may impede children’s ability to develop awareness of hunger and satiety [9]. By focusing on external cues, children being pressured to eat may perceive food as less desirable, while children whose parents restrict amount of food may perceive it as more desirable [10,12]. It is also possible that parents engage in these feeding practices when they are concerned about their children’s weight. Several longitudinal studies have begun to address the causal direction of these relationships, but findings have been inconsistent [12,57-59].

Fathers’ greater use of food to control their child’s behavior was related to children’s lower weight status. This feeding behavior, encompassing use of food as a reward and use of food to control emotions, is considered to be evidence of parental use of control over child feeding and was originally conceptualized as an aspect of restriction [20]. However, use of food to control child’s behavior may reduce the desirability of food for children, as suggested by the finding that it was associated with lower child weight status. In contrast, restriction of food appears to increase the desirability of food [9]. Other research has also found that reward and restriction are two separate constructs [3]. Accordingly, we suggest that parental restriction of food and use of food to control behavior be measured separately in future research.

Although fathers play an important and unique role in families [60], few studies of parental feeding practices have included fathers. In the current study, we found both similarities and differences between mothers and fathers. Mothers’ and fathers’ feeding practices were associated, but fathers reported using pressure to eat and using food to control behavior more often than mothers, and mothers reported more positive involvement in children’s eating. Both mothers’ and fathers’ feeding practices were linked to children’s weight, but use of food to control behavior was linked to children’s lower weight only for fathers, while greater positive involvement in children’s eating was linked to children’s lower weight only for mothers. These results hint that compared to mothers, fathers may engage in more feeding practices that reflect control over child feeding. Further research in Mexican American families that examines culturally-related parental roles may help to illuminate these findings. In addition, future research on parental feeding practices that is conducted in any cultural group could benefit from including fathers.

This study has several limitations. Because the research was cross-sectional, it was not possible to determine whether parental feeding practices influence children’s weight status, or whether parents’ feeding practices are responses to concerns about children’s weight. The findings from this research cannot be generalized beyond Mexican American families with mostly immigrant parents. It is also possible that the findings of this study are due to participants’ SES or immigrant status, rather than their membership in a particular cultural group; consequently, these findings may apply to other cultural or ethnic groups. In addition, because we studied only children ages 8–10, we cannot generalize beyond this age range. Thus, future research could test the appropriateness of the PFP for use in other populations and with a broader age range of children. Another limitation is that although the alphas for the second-order factors were all acceptable, several first-order factors that contained few items had low alphas. Future research using this scale could perform further developmental work, such as focus groups or individual qualitative interviews, to create additional items for those subscales. An additional limitation is the possibility that parents influenced one another’s responses. However, because the interviews were lengthy, it would have been difficult for participants to remember their partner’s responses. Finally, our understanding of the parental feeding practices identified in this research would be enhanced by additional sources of validity, such as observations of children’s mealtimes.

## Conclusions

This study reported on the reliability and validity of the Parental Feeding Practices (PFP) Questionnaire, a new measure designed for use with Mexican American parents. Four parental feeding practice dimensions were identified. Dimensions had equivalent meaning for mothers and fathers, but mothers and fathers differed somewhat in their use of feeding practices. All four feeding practice dimensions were related to children’s BMI percentiles, for one or both parents.

Our results underscore the importance of using qualitative methods when developing or adapting measures. Not only do qualitative approaches such as focus groups yield new items, but also concepts and behaviors that are culturally congruent can be identified. Moreover, cognitive interviewing is useful for examining the acceptability of both new items and items from existing measures. Using qualitative methods in this study resulted in a comprehensive measure of parental feeding practices among Mexican Americans, which provides a more complete assessment of parents’ positive involvement in their children’s eating than previous measures. It also distinguishes among restriction of amount of food, monitoring/limiting high-calorie foods, and use of food to control behavior. Using similar qualitative approaches, investigators could adapt the PFP for other cultural groups. Because of high rates of obesity among Mexican American children, it is imperative that parental feeding practices be measured accurately. This culturally-based measure can be utilized in future research assessing parental feeding practices in Mexican American families, and could inform interventions targeting obesity among Latino children.

## Abbreviations

BMI: Body mass index; PFP: Parental Feeding Practices Questionnaire.

## Competing interests

The authors declare that they have no competing interests.

## Authors’ contributions

All authors were involved in all parts of the study, contributed to conceptualization of the manuscript, participated in questionnaire development, and approved the final manuscript. JT was responsible for the study design and drafted the manuscript. SG and CdG performed statistical analyses and SG wrote much of the statistical analysis and results sections. CP was responsible for overseeing the translation of the questionnaire and the study implementation. LP, EF, and JD contributed to the writing, interpretation of the results, and finalizing of the manuscript. LG provided access to participants and obtained ethics approval. NB reviewed the study design and provided guidance on measuring body mass index.

## Supplementary Material

Additional file 1 Appendix. Click here for file
