# Supplementary material for: Parental feeding practices in Mexican American families: initial test of an expanded measure
Source: Int J Behav Nutr Phys Act. 2013 Jan 17;10:6. doi: 10.1186/1479-5868-10-6 (PMC3552721; doi:10.1186/1479-5868-10-6)
Supplement: Additional file 1 — Appendix. [file 1479-5868-10-6-S1.docx]

**Appendix**

Psychometric analysis plan

The PFP measurement model was assessed in six stages. Initially, we fit a model to test the equivalence of mothers' and fathers' PFP item mean vectors and covariance matrices. Using one record per couple and 136 (68×2) variables, we first estimated the PFP item mean vector and covariance matrix. The *equal moments* model (Model 1) imposed cross-parent equality constraints on corresponding elements of the PFP item mean vector and the intra-parent elements of the PFP covariance matrix. All inter-parent covariances of PFP items were freely estimated. The results suggested equivalence of PFP item mean vectors and covariance matrices across mothers and fathers, $\bar{\chi}_{\text{S-B}}^{2}$= 2060.90 with 2079 degrees of freedom, RMSEA=0, CFI=1.0. That finding suggested that if a suitable factor model could be identified, then its parameters would be invariant across mothers and fathers.

Stages two through four sought and tested a first-order factor model of the PFP items. In stage two, we submitted the stacked mothers' and fathers' data to an oblique principal components cluster analysis of the 68 PFP items [43]. Solutions with 1 to 20 clusters were generated and evaluated, in a largely subjective manner, to select the solution that best balanced parsimony with conceptual distinctiveness of item clusters. We selected the 14-cluster solution. Based upon VARCLUS results, two items were dropped because they were judged to share insufficient substantive content with the other items [#23: 'How often do you withhold sweets/dessert from your child in response to bad behavior?' and #29: 'How often do you help your child to eat dinner (for example, cutting the food into smaller pieces)?']. The 14-cluster solution from stage 2 implied a first-order factor model where each cluster represented a common factor that was identified by its respective items (i.e., no cross-factor loadings). In stage three, a series of initial first-order confirmatory factor analyses (CFA) were fit to the stacked data, starting with smaller models of individual clusters/factors and then gradually combining clusters/factors into multi-factor models [61] (results not shown). During this process, an additional three items were dropped, because of high factor cross-loadings or low primary factor loadings (#58: 'How often do you ask your child questions about the food during dinner?'; #67: 'How often do you let your child decide how much he/she needs to eat?'; #68 'If your child thinks he/she doesn't like a certain food, how often do you give him/her a small serving of it?'). Therefore, 63 of the original 68 PFP items were retained. The 63 items and their corresponding first-order factors (clusters) are listed in Table 2 of the manuscript. In stage four, the data were reshaped to have one record per couple. The fit of the 63 item first-order factor model as well as its invariance across mothers and fathers was assessed with a pair of nested CFA models. A *first-order configural invariance* model simultaneously estimated parameters of separate, but correlated CFA models for mothers and fathers. In this model the configuration of items and factors was the same for mothers and fathers, corresponding item residual variances were allowed to covary across mothers and fathers, and all factor variances and covariances were allowed to covary across mothers and fathers. For purposes of model identification, one factor loading per factor was fixed to equal unity and the corresponding item intercept was fixed to equal zero. No equality constraints were imposed across mothers' and fathers' model parameters. The subsequent *first-order strict invariance* model was identical to the first-order configural invariance model, except that equality constraints were imposed on corresponding parameter estimates across the mothers' and fathers' parts of the model: factor loadings, item intercepts, and item residual variances, as well as factor variances and covariances. We note that equivalence of factor variances and covariances across modeled groups is not a condition of strict factorial invariance; however, we added those constraints to force estimated factor loadings to be equal for mothers and fathers. Usually, a series of intermediate factorial invariance models are also tested (e.g., 'factor pattern' or 'metric' invariance; 'strong factorial' or 'scalar' invariance; [50-52] but the excellent fit of the equal moments model obviated the need to consider them.

Stages five and six examined a second-order factor model of the PFP items. In stage five, we sought a parsimonious second-order factor structure by creating summated scales corresponding to each first-order factor and submitting those data to an exploratory factor analysis with oblique rotation. As noted in the main text, the 14 first-order factor scale scores had a complex factor structure, resulting in subsequent empirical estimation problems for second-order CFA models. The second-order factor structure was greatly simplified by dropping 2 first-order factors, 'Allow child to choose meal menu' and 'Allow child to leave table without finishing meal,' resulting in a second-order factor model with 12 first-order factors and four second-order factors. In stage six, the fit of the second-order factor model and its invariance across mothers and fathers was tested with a pair of nested CFA models. A *second-order configural invariance* model estimated separate, but correlated CFA models for mothers and fathers. In this model, the groupings of items within first-order factors as well as first-order factors within second-order factors was the same for mothers and fathers, corresponding item residual and first-order factor residual variances were allowed to covary across mothers and fathers, and all second-order factor variances and covariances were allowed to covary across mothers and fathers. For purposes of model identification, one first-order factor loading and one second-order loading per factor was fixed to equal unity and the corresponding item and first-order factor intercepts were fixed to equal zero. No equality constraints were imposed across mothers' and fathers' model parameters. Next, a *second-order strict invariance model* additionally imposed equality constraints across mothers' and fathers' corresponding first- and second-order factor loadings, item and first-order factor intercepts, item and first-order factor residual variances, as well as second-order factor variances and covariances.

In analysis stages 1, 3, 4, and 6, models were fit to the data via maximum likelihood (ML) with LISREL 9.0 beta version [44]. In stage 6, LISREL's modification indices were consulted when considering whether to free cross-loadings in the second-order factor structure. Because the empirical item distributions were non-normal, the Satorra-Bentler scaled chi-square, $\chi_{\text{SB}}^{2}$, and robust parameter standard errors were estimated [45]. The weight matrix required to compute these adjustments was estimated using the approach described by [62] via 10,000 bootstrap samples. Goodness of fit was assessed by examining model chi-squares and degrees of freedom, the root mean square error of approximation (RMSEA [46]), and the comparative fit index (CFI [47]). Generally, RMSEA values below .05 or .06 and CFI values above .95 suggest approximate model fit [48-49].

Although <1% of all data values were missing, we accommodated missing values via multiple imputation (MI) and the expectation-maximization (EM) algorithm [40-42]. The MI and EM algorithms assumed that values were missing at random (MAR), conditional on observed values [40, 42, 63]. In stages 2 and 5, we fit models to the EM item (or scale) covariance matrix, estimated from the stacked data. In stages 1, 3, 4, and 6, missing values were imputed using SAS PROC MI [43]; substantive models were each fit to five multiply imputed data sets. For each model fit to multiply imputed data, parameter estimates were aggregated across solutions using Rubin's rules [41].

Technically, the ML chi-square goodness of fit statistics can be combined across imputed data sets resulting in a single *F*-test [64]. However, the resulting *F*-tests displayed poor type 1 error control in modeling stages 2-6: each chi-square statistic was highly significant, yet all corresponding *F*-tests were non-significant. Based upon simulation results, [64] Li found that their *F*-test became more conservative as the fraction of missing information approached zero and as the number of model parameters increased. Their simulation included conditions with the fraction of missing information as small as 0.1 and the number of model parameters as large as 25. In comparison, the current application entails an average item-wise fraction of missing information <0.01 and models with several hundred parameters. This may explain why the Li et al *F*-test was highly conservative in the current application. Furthermore, to our knowledge a corresponding *F*-test has not been developed for combining Satorra-Bentler scaled chi-squares. Therefore, as a descriptive point of reference, for each model we report chi-square statistics averaged across imputed data sets, with model degrees of freedom. RMSEA and CFI values for each model also were averaged across imputed data sets.

**Appendix References**

**(Note: references 1 – 60 are provided at the end of the article)**

61. Jöreskog KG: **Testing structural equation models.** In *Testing structural equation models.* Edited by Bollen KA, Long JS. Newbury Park, CA: Sage Publications; 1993

62. Yung YF, Bentler PM: **Bootstrap-corrected ADF test statistics in covariance structure analysis.** *Br J Math Stat Psychol* 1994, **47 ( Pt 1):**63-84.

63. Rubin D: **Inference and missing data.** *Biometrika* 1976, **63:**581-592.

64. Li KH, Meng XL, Raghunathan TE, Rubin DB: **Significance Levels from Repeated P-Values with Multiply-Imputed Data.** *Stat Sinica* 1991, **1:**65-92.

Table 5. Correlations, means, and standard deviations of first-order feeding practices scales (mothers below the diagonal: fathers above the diagonal: correlations between mothers and fathers on the diagonal).

|  | **(1)** | **(3)** | **(7)** | **(12)** | **(14)** | **(2)** | **(9)** | **(5)** | **(10)** | **(4)** | **(6)** | **(13)** | **(8)** | **(11)** |
| --- | --- | --- | --- | --- | --- | --- | --- | --- | --- | --- | --- | --- | --- | --- |
| **Positive involvement in child eating** |  |  |  |  |  |  |  |  |  |  |  |  |  |  |
| **(1)** Monitor/limit high-calorie foods | **.22*** | .62* | .31* | .42* | .24* | .19 | .12 | -.03 | -.10 | .32* | -.12 | .49* | .24* | .12 |
| **(3)** Encourage /compliment healthy eating | .52* | **.24*** | .51* | .47* | .22* | .36* | .18 | .16 | .09 | .21* | .02 | .33* | .47* | .17 |
| **(7)** Encourage a variety of new foods | .35* | .45* | **.10** | .37* | .28* | .32* | .27* | .16 | .19 | .10 | .10 | .25* | .31* | .09 |
| **(12)** Ask child what he/she ate | .44* | .45* | .17 | **.24*** | .29* | .12 | .13 | .05 | -.01 | .22* | -.14 | .34* | .29* | .20* |
| **(14)** Provide small servings | .26* | .27* | .33* | .18 | **.20*** | .28* | .30* | .19 | .21* | .17 | .02 | .22* | .18 | -.02 |
| **Pressure to eat** |  |  |  |  |  |  |  |  |  |  |  |  |  |  |
| **(2)** Tell child to eat all food on plate | .14 | .43* | .11 | .15 | .08 | **.35*** | .55* | .32* | .45* | -.05 | .07 | .12 | .05 | -.21* |
| **(9)** Require child to eat even if not hungry | .03 | .23* | .14 | -.04 | .08 | .56* | **.42*** | .28* | .41* | -.12 | .05 | -.00 | -.00 | -.25* |
| **Use of food to control behavior** |  |  |  |  |  |  |  |  |  |  |  |  |  |  |
| **(5)** Use food to control emotions | -.14 | .23* | -.05 | .03 | .07 | .42* | .37* | **.14** | .43* | .14 | .17 | .12 | .13 | -.03* |
| **(10)** Use food as reward | -.02 | .13 | .10 | -.06 | -.03 | .35* | .24* | .43* | **.24*** | -.10 | .12 | .07 | .10 | -.18 |
| **Restriction of amount of food** |  |  |  |  |  |  |  |  |  |  |  |  |  |  |
| **(4)** Encourage child to eat less | .18 | .10 | -.05 | .21* | .08 | -.04 | -.25* | -.05 | -.12 | **.59*** | -.19 | .43* | .02 | .14 |
| **(6)** Allow child to control snacking and second servings | -.13 | .03 | .18 | -.13 | .12 | -.07 | .14 | .11 | .08 | -.29* | **.14** | -.21* | .18 | .19 |
| **(13)** Limit eating between meals | .39* | .30* | .16 | .20* | .16 | .08 | .05 | -.06 | .02 | .40* | -.37* | **.20*** | .09 | .19 |
| **N/A** |  |  |  |  |  |  |  |  |  |  |  |  |  |  |
| **(8)** Allow child to choose meal menu | .08 | .31* | .13 | .02 | .14 | .04 | .10 | .26* | .22* | -.10 | .34* | -.12 | **.13** | .25* |
| **N/A** |  |  |  |  |  |  |  |  |  |  |  |  |  |  |
| **(11)** Allow child to leave table without finishing meal | .06 | .09 | .16 | .11 | .12 | -.22* | -.23* | -.07 | -.09 | .24* | .08 | .14 | .22* | **.29*** |
|  |  |  |  |  |  |  |  |  |  |  |  |  |  |  |
|  |  |  |  |  |  |  |  |  |  |  |  |  |  |  |
| **Means (SD)** |  |  |  |  |  |  |  |  |  |  |  |  |  |  |
|  |  |  |  |  |  |  |  |  |  |  |  |  |  |  |
| Mothers | 3.88 ^a^ (.76) | 3.46 (.76) | 3.36 ^b^ (1.06) | 3.66 (1.06) | 2.67 ^c^ (.91) | 2.51^d^ (.93) | 2.05 (.93) | 1.37 (.54) | 1.67 (.56) | 1.94 (.87) | 2.44 (.67) | 2.57 (.86) | 2.53 (.77) | 2.67 (.87) |
|  |  |  |  |  |  |  |  |  |  |  |  |  |  |  |
| Fathers | 3.68 ^a^ (.93) | 3.38 (.84) | 3.10 ^b^ (1.10) | 3.07 (1.15) | 2.35 ^c^ (.95) | 2.73 ^d^ (.93) | 2.12 (1.01) | 1.47 (.55) | 1.76 (.65) | 1.94 (.83) | 2.43 (.68) | 2.52 (.84) | 2.43 (.76) | 2.56 (.84) |

^*^ *p* <.01.

^a, b, c, d^ Means sharing a common superscript are significantly different, *p* <.05.

Table 6. Partial correlations between first-order parental feeding practices scales and children’s BMI percentiles, controlling for parents’ acculturation, occupational status, and parents’ BMI.

Children’s BMI

|  | Mothers | Fathers |
| --- | --- | --- |
| **(1) Positive involvement in child eating** |  |  |
| (1) Monitor/limit high-calorie foods | -.01 | .18* |
| (3) Encourage /compliment healthy eating | -.24** | -.10 |
| (7) Encourage a variety of new foods | -.21** | -.13 |
| (12) Ask child what he/she ate | -.07 | -.03 |
| (14) Provide small servings | -.11 | -.03 |
| **(2) Pressure to eat** |  |  |
| (2) Tell child to eat all food on plate | -.22** | -.27*** |
| (9) Require child to eat even if not hungry | -.33*** | -.30*** |
| **(3) Use of food to control behavior** |  |  |
| (5) Use food to control emotions | -.15* | -.11 |
| (10) Use food as reward | -.01 | -.21** |
| **(4) Restriction of amount of food** |  |  |
| (4) Encourage child to eat less | .49*** | .46*** |
| (6) Allow child to control snacking and second servings | -.20** | -.14 |
| (13) Limit eating between meals | .19* | .24** |
| **N/A** |  |  |
| (8) Allow child to choose meal menu | -.19* | -.07 |
| **N/A** |  |  |
| (11) Allow child to leave table without finishing meal | .06 | .20** |

* p < .05, ** p < .01, *** p < .001.
